# Survival and predictors of mortality among human immunodeficiency virus patients on anti-retroviral treatment at Jinka Hospital, South Omo, Ethiopia: a six years retrospective cohort study

**DOI:** 10.4178/epih.e2016049

**Published:** 2016-11-06

**Authors:** Erdaw Tachbele, Gobena Ameni

**Affiliations:** 1 Department of Nursing and Midwifery, Addis Ababa University College of Health Sciences, Addis Ababa, Ethiopia; 2 Aklilu Lemma Institute of Pathobiology, Addis Ababa University, Addis Ababa, Ethiopia

**Keywords:** Human immunodeficiency virus, Antiretroviral therapy, Survival, Mortality, Ethiopia

## Abstract

**OBJECTIVES:**

The survival rate of human immunodeficiency virus (HIV)-infected patients receiving treatment in Ethiopia is poorly understood. This study aimed to determine the survival rate and predictors of mortality among HIV-infected adults on antiretroviral therapy (ART) at Jinka Hospital, South Omo, Ethiopia.

**METHODS:**

A 6-year retrospective cohort study was conducted using 350 patient records drawn from 1,899 patients on ART at Jinka Hospital from September 2010 to August 2015. The data were analyzed using Kaplan-Meier statistics and Cox regression models.

**RESULTS:**

Of the 350 study participants, 315 (90.0%) were censored and 35 (10.0%) died. Twenty-two (62.9%) of the deaths occurred during the first year of treatment. The total follow-up encompassed 1,995 person-years, with an incidence rate of 1.75 deaths per 100 person-years. The mean survival time of patients on highly active antiretroviral therapy (HAART) was 30.84±19.57 months. The overall survival of patients on HAART was 64.00% (95% confidence interval [CI], 61.85 to 66.21%) at 72 months of follow-up. The significant predictors of mortality included non-disclosure of HIV status (adjusted hazard ratio [aHR], 5.82; 95% CI, 1.91 to 17.72), a history of tuberculosis (aHR, 1.82; 95% CI, 1.41 to 3.51), and ambulatory (aHR, 2.97; 95% CI, 1.20 to 8.86) or bedridden (aHR, 4.67; 95% CI, 1.30 to 17.27) functional status, World Health Organization (WHO) clinical stage IV illness (aHR, 24.97; 95% CI, 2.75 to 26.45), and substance abusers (aHR, 3.72; 95% CI, 1.39 to 9.97).

**CONCLUSIONS:**

Patients with a history of tuberculosis treatment, ambulatory or bedridden functional status, or advanced WHO clinical stage disease, as well substance abusers, should be carefully monitored, particularly in the first few months after initiating antiretroviral therapy. Patients should also be encouraged to disclose their status to their relatives.

## INTRODUCTION

Human immunodeficiency virus (HIV) and acquired immunodeficiency syndrome (AIDS) have created enormous challenges worldwide. Since 2000, around 38.1 million people have become infected with HIV and 25.3 million people have died of AIDS-related illnesses [1].

Despite significant global achievements in all aspects of controlling this pandemic, HIV/AIDS remains a major public health concern in sub-Saharan Africa, which is where 25.8 million (70%) of the 36.9 million people estimated to be infected by HIV in 2014 live [1].The region accounts for 1.4 million (70%) of new infections globally as of the end of 2014 [2].

Introduced in 1996, highly active antiretroviral therapy (HAART) was a breakthrough in the developed world, leading to the reduction of mortality and improvements in the quality of life of people living with HIV and AIDS. The introduction of antiretroviral drugs also significantly lowered the rate of HIV transmission from mother to child [3,4]. Thus, antiretroviral therapy (ART) has become an integral part of the continuum of HIV care. As a result, the number of people living with HIV continues to increase [5,6].

As of June 2015, 15.8 million people living with HIV had access to ART, up from 13.6 million in June 2014 [2]. According to the Joint United Nations Programme on HIV/AIDS, the world has arrested and reversed the spread of HIV. New HIV infections and AIDS-related deaths have decreased significantly since the peak of the epidemic, which has been expected to lead to the end of the AIDS pandemic by 2030 [2].

The HIV/AIDS epidemic has remained one of the important public health challenges in Ethiopia since it was first recognized in 1984. Currently, Ethiopia has a low rate of HIV endemicity, with a national prevalence of 1.1% [7]. Currently, 753,100 people are living with HIV in Ethiopia, and the national HIV prevalence has declined from 1.5% in 2011 to 1.1% in 2015 (2014 Estimation and Projection Package and Spectrum software).

The government of Ethiopia launched its ART initiative in 2003 based on a subsidized fee-based approach. Subsequently, the service was rapidly scaled up with a number of global and national initiatives [8]. With the help of concerted global and national actions, ART became available free of charge in 2005 in the country. The ART program was significantly scaled up by decentralizing it to health centers in 2006, and implementing a nationwide campaign to achieve national targets both for ART and for HIV testing and counseling, as recommended by the World Health Organization (WHO) [9-11].

Currently, 339,043 adults are receiving ART in the country, corresponding to an ART coverage rate of 65%. The ART regimens in Ethiopia include stavudine (D4T), lamivudine (3TC), nevirapine (NVP), zidovudine (AZT), 3TC-NVP, D4T-3TC-efavirenz (EFV), and AZT-3TC-EFV [12].

The eligibility criteria for initiating ART have been frequently revised. Until the end of 2012, the eligibility criterion for initiating ART was a CD4 count of less than 200 cells/μL of blood or WHO stage IV disease. Since 2013, all patients with CD4 counts less than 350 cells/μL of blood, or WHO stage III or IV disease are eligible for ART initiation. Through the end of 2014, the WHO recommended starting ART in patients with a higher CD4 count (up to 500 cells/μL of blood), in order to help countries improve access to ART and to increase its utilization [13]. Very recently, the WHO announced a new recommendation to initiate ART in all adults, adolescents, and children with HIV, regardless of CD4 count or disease stage [14].

Despite the scale-up of ART, retention of patients and early mortality remain major challenges for such programs in sub-Saharan Africa [15]. Several retrospective and prospective cohort studies have been conducted in different parts of Ethiopia to investigate survival and the determinants of mortality in adult HIV/AIDS patients after the initiation of ART. The estimated average mortality rates ranged from 8.5 to 12.0%, with the majority of deaths occurring in the first year of treatment. The main predictors of mortality were found to be WHO stage III and IV disease, anemia, poor ART adherence, a CD4 count <200 cells/μL of blood, not taking cotrimoxazole for prophylaxis, low hemoglobin levels, bedridden functional status, chronic diarrhea, opportunistic infections, educational status, place of residence, and nutrition [9,16-24].

However, the factors contributing to the survival and mortality of HIV-infected patients on ART have not been explored in the area of the current study: South Omo, southern Ethiopia. This area is unique because it is mainly inhabited by pastoralist and agro-pastoralist populations, including more than 16 ethnic groups with lifestyles and cultures different from those of the rest of the country. As a result of its diverse ethnic groups and cultures, South Omo is a tourist destination in Ethiopia. Hence, this study was designed to investigate the outcomes of ART, focusing on survival and predictors of mortality among HIV-infected adults on ART in Jinka Zonal Hospital, South Omo, Ethiopia.

## MATERIALS AND METHODS

### Study design, area, and period

A facility-based retrospective cohort study design was conducted in Jinka Hospital, Jinka, from November to March 2016. Adult patients who started HAART from September 2010 to August 2015 in this hospital were recruited for the study. Jinka, the administrative capital of the South Omo Zone in the Southern Peoples, Nations, and Nationalities Region, is located 750 km to the south of Addis Ababa, the capital of Ethiopia. The hospital provides general outpatient and inpatient care, surgical and obstetric emergency services, and acts as the only zonal referral hospital for all health services for the pastoralist and agro-pastoralist population residing in South Omo. Jinka Zonal Hospital started providing ART services to HIV/AIDS patients in October 2005. As of August 2015, a total of 3,199 HIV/AIDS patients have utilized ART services at this hospital, of whom 1,899 are currently on ART.

### Study population

The records of all HIV-positive adults who were on ART at Jinka Zonal Hospital and who enrolled in treatment from September 1, 2010 to August 30, 2015 were the source population. The records of selected HIV-positive adults who had started ART at the hospital within this period were included in the study.

### Ethical considerations

Ethical clearance was obtained from the institutional review board of the Aklilu Lemma Institute of Pathobiology, Addis Ababa University, and Jinka Hospital officials received a support letter from the university. Retrospective data were collected anonymously and confidentially.

### Sample size determination

The sample size was estimated using the power and sample size calculation software developed by Dupont & Plummer [25]. To obtain the optimum sample size, two populations were considered, categorized by WHO staging as the main exposure variable for HIV/AIDS-related deaths during the 6-year follow-up period. The calculations were based on the following assumptions: a 5% level of significance (two-sided), a power of 80%, and a ratio of exposed to non-exposed of 2:1. The estimated median survival time was 60 months for the non-exposed group (WHO stages I and II) and 40 months for the exposed group (WHO stages III and IV) [19]. Accordingly, the calculated sample size was 319 (213 in WHO stages III and IV, and 106 in stages I and II). After adding 10% as a contingency modifier, the total sample size was 350 (220 for exposed subjects and 130 for non-exposed subjects).

### Sampling procedure

Simple random sampling was done using commands in Excel (Microsoft Corp., Redmond, WA, USA); all 1,899 records of patients receiving ART in Jinka Hospital were listed in a single Excel spreadsheet, and a random selection of 350 records was made.

### Data collection methods

The data were collected using a standard checklist on an Excel spreadsheet, incorporating information extracted from electronic and paper-based ART registration and follow-up forms used in the ART clinic. Using the data extraction sheet, information about study participants, such as socio-demographic characteristics, residence, weight, disclosure status, duration on ART (in months), functional status, types of opportunistic infections, baseline WHO clinical staging, baseline and follow up CD4 cell counts, ART regimen type, ART eligibility criteria, substance use, regimen changes and reasons for those changes, and survey endpoints were retrieved from the clinical records of the HIV/AIDS patients by trained ART data clerks. Incomplete clinical records were omitted and were replaced with the record of the next patient on the list. The data collection form was checked for completeness and consistency with each patient’s clinical records by a supervisor, a public health officer by profession, on a daily basis.

### Independent and outcome variables

The primary outcome variable was time to event in months. HAART-initiated patients were followed until the date of death, loss to follow-up, transferring out, or the end of the study. Individuals who were on HAART, lost to follow-up, or had transferred out at the end of the study period were censored; that is, they were considered to be alive for the time period that they had been under follow-up. The survival time was calculated in months using the time between the dates of treatment initiation and the date of the event (death) or date of censoring. The predictor variables were age, sex, educational level, marital status, occupation, residence, functional status, WHO clinical stage, drug regimen, CD4 count, substance use, and opportunistic infections.

### Data entry and analysis

After the Excel data were reshaped and adjusted, the data were transported to SPSS version 20.0 (IBM Corp., Armonk, NY, USA). Descriptive statistics such as median, interquartile range (IQR), and mean and standard deviation (SD) were used to summarize the characteristics of the cohort. The Kaplan-Meier log-rank model was used to estimate the survival time of ART patients based on the independent variables. Bivariate and multivariate Cox proportional hazards regression models were used to identify the predictors of mortality. Variables found to be statistically significant in the bivariate analysis (p<0.05) were included in the multivariable Cox proportional hazards regression model to identify independent predictors of mortality and to estimate the adjusted hazard ratios (aHRs). Both crude hazard ratios and aHRs with 95% confidence intervals (CIs) were reported. Variables that were statistically significant (p<0.05) were considered predictors of mortality among the study participants.

## RESULTS

The study included 350 ART patients who were followed up for a total of 72 months, with a median of 28.5 months. The majority of patients (59.1%) were female; 206 (58.9%) were married; 204 (58.3%) were illiterate, whereas 108 (30.9%) had completed primary school; 247 (70.6%) were Orthodox Christians; 247 (70.6%) were urban dwellers; 114 (32.6%) and 78 (22.3%) were daily laborers and farmers, respectively; 245 had four or fewer family members in their household; 318 (90.8%) had an unimpaired functional status; 330 (94.3%) had disclosed their HIV status; 313 (89.4%) had at least one opportunistic infection; and 183 (52.3%) were in WHO clinical stage III. The median age, CD4 count, and weight of the patients at base line were 30 years (IQR, 25 to 36 years), 254 cells/μL (IQR, 149 to 334 cells/μL), and 52 kg (IQR, 47 to 59 kg), respectively. Of the 350 study participants, 315 (90.0%) were censored, including 277 (79.1%) on ART, 21 (6.0%) who were lost to follow-up, and 17 (4.8%) who transferred. The remaining 35 (10.0%) died. Twenty-two (62.9%) of the deaths occurred during the first year of treatment, while eight (22.9%) deaths occurred in the second year of follow-up.

The socio-demographic and clinical characteristics of the study participants are given in Tables 1 and 2, respectively.

### Descriptive analysis

**Figure 1.**
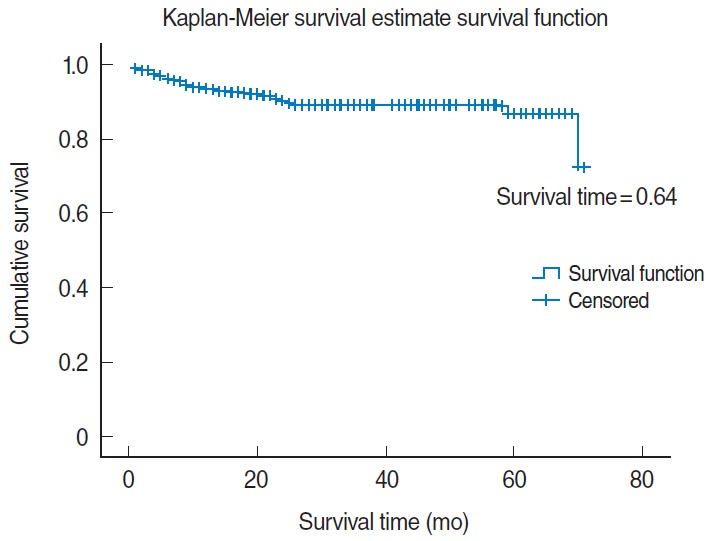
Overall survival probability of patients starting antiretroviral therapy in Jinka Hospital from September 2010 to August 2015.

The total extent of follow-up was 1,995 person-years, with an incidence rate of 1.75 deaths per 100 person-years. The mean survival time of patients on HAART was 30.84 months (SD, 19.57 months). The overall survival of patients on HAART was 64.00% (95% CI, 61.85 to 66.21%) at 72 months of follow-up (Figure 1).

The log-rank test was conducted to check for the existence of any significant differences in survival among various levels of the categorical variables considered in the study. Accordingly, the Kaplan-Meier analysis indicated significant evidence of differences in survival times in the categories of past tuberculosis treatment, functional status, WHO clinical staging, baseline CD4 count, initial regimen types, substance abuse, disclosure status, and opportunistic infections (Table 3).

The Kaplan-Meier survival function (Table 3) indicated a significantly higher survival of HAART patients with the baseline characteristics of clinical stage I and II disease (67.12%; 95% CI, 64.34 to 69.89%), unimpaired functional status (66.27%; 95% CI, 64.39 to 68.17%), HIV status disclosure (64.96%; 95% CI, 62.83 to 67.10%), baseline CD4 count of at least 351 cells/μL (68.84%; 95% CI, 65.91 to 71.76), and no substance abuse (68.34%; 95% CI, 66.25 to 70.43).

The lowest survival probabilities were observed for patients with a bed ridden functional status (13.25%), a history of tuberculosis treatment (50.86%), no disclosure of HIV status (52.41%), a baseline CD4 count <350 cells/μL (52.83%), and WHO stage IV disease (43.7%) (Table 3).

**Figure 2.**
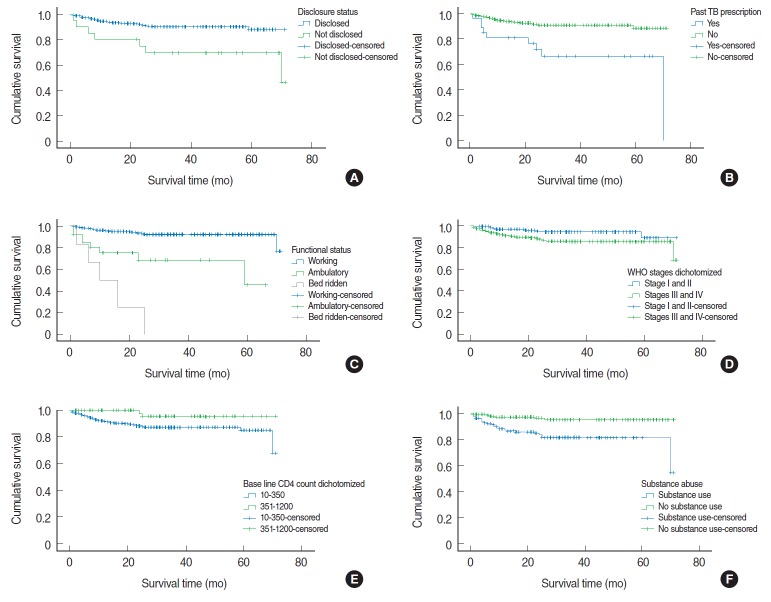
Survival outcomes (Kaplan-Meier analysis) by disclosure status (A), past tuberculosis (TB) treatment (B), functional status (C), WHO staging (D), baseline CD4 cell count (E), and substance abuse (F) among HIV patients receiving antiretroviral therapy at Jinka Hospital, South Omo, Ethiopia, 2016 (n=350). WHO, World Health Organization; HIV, human immunodeficiency virus.

To identify independent predictors of mortality and survival, multivariate regression was performed for all predictors found to be significantly associated with survival in the bivariate analysis. According to the Cox survival regression model, all predictors associated in the bivariate analysis were found to be strong predictors of mortality in the multivariate analysis, except for baseline CD4 cell count (Figure 2, Table 4). The HR for death was 5.82 (95% CI, 1.91 to 17.72) in patients who had not disclosed their status, compared to those who had; 1.82 (95% CI, 1.41 to 3.51) in patients with a history of tuberculosis treatment; 2.97 (95% CI, 1.20 to 8.86) and 4.67 (95% CI, 1.30 to 17.27) in patients with an ambulatory and bedridden functional status, respectively, in comparison those with an unimpaired functional status; 24.97 (95% CI, 2.75 to 26.45) in patients with WHO clinical stage IV disease; and 3.72 (95% CI, 1.39 to 9.97) in patients reporting substance abuse. However, the HR for mortality was found to be reduced by 0.20 (95% CI, 0.07 to 0.53) in patients taking the 1e (TDF-3TC-EFV) regimen in comparison to the other five regimens used (Table 4).

The ART retention rate (79.0% after 72 months of follow-up) was significantly associated with occupation and WHO clinical stage, with housewives (adjusted odds ratio [aOR], 3.34; 95% CI, 2.08 to 18.48) and daily laborers (aOR, 2.60; 95% CI, 1.22 to 5.52) more likely to continue ART than other occupation types (Table 5).

## DISCUSSION

### Retention rate

The retention rate of 79.0% after 72 months of follow-up in this study is comparable with studies performed throughout most of sub-Saharan Africa, where retention ranges have been reported to range from 57 to 72% [15,26]. Nearly 80.0% (95% CI, 76.7 to 82.1%) of patients were retained in care in the first 3.5 years of ART in Addis Ababa [24], while a value of 64.7% has been reported for in southern Ethiopia [27], which is lower than what was found in the current study. According to Mekuria et al. [24], severe immune deficiency at enrollment in care or at the time of ART initiation, as well as a bedridden or ambulatory functional status at the start of ART, predicted attrition in Addis Ababa. Similar to our findings, a 74.0% retention rate was reported in another large-scale retrospective study done in Ethiopia [21]. Retention was significantly associated with occupation and WHO clinical stage. Housewives (aOR, 3.34; 95% CI, 2.08 to 18.48) and daily laborers (aOR, 2.60; 95% CI, 1.22 to 5.52) were more likely to be retained in the ART program than other occupation types. Patients with WHO stage I (aOR, 6.20; 95% CI, 2.08 to 18.48) and II (aOR, 5.02; 95% CI, 1.94 to 10.53) disease were more likely to be retained in the ART program than patients with an advanced WHO stage of disease.

### Probability of survival and rate of mortality

In this study, the overall survival probability of HIV/AIDS patients on ART was found to be 64.00% (95% CI, 61.85 to 66.21%) at 72 months of follow up, which is slightly higher than was reported in studies performed in Debre-Markos, Addis Ababa, and Cameroon, where the mean survival probabilities were 57% (95% CI, 53 to 60%) [19], 41.00% (95% CI, 39.69 to 42.64%) [28], and 47% (95% CI, 40 to 55%) [29], respectively, but similar to a study carried out in Gonder, Ethiopia, in which 61.4% of the patients survived and 10.4% died [16].

In this 6-year retrospective cohort study, 35 patients died of a total of 350 participants over six years of follow-up, resulting in a total death prevalence of 10.0% and an incidence rate of 1.75 per 100 person-years. Twenty-two (62.9%) of the deaths occurred during the first year of follow-up. The total number of deaths that occurred in this study is much lower than was reported in a study conducted in Debre-Markos Hospital, Ethiopia, where 40.7% of patients died [18]. Moreover, data from Senegal, Malawi, and Tanzania have indicated high rates of HIV-related mortality, ranging from 24.2 to 44.0% [30], in contrast to the present study.

However, our results are similar to the mortality rate of 11.1% exhibited in the Somali region of Ethiopia [22], and with other findings from sub-Saharan Africa, where between 8 and 26% of patients die in the first year of ART, with most deaths occurring in the first few months [31]. Although the immunological and virological responses to ART in patients in sub-Saharan Africa are comparable to the responses in patients treated in high-income countries, early mortality rates in the region have remained very high [31]. The main reasons for early mortality have been found to be baseline CD4 cell counts less than 50 cells/μL, advanced WHO stage, and opportunistic infections. Hence, the earlier diagnosis of HIV infection, timely initiation of antiretroviral treatment, and strengthening of the HIV care continuum should be implemented in order to reduce early mortality among HIV/AIDS patients.

The multivariate Cox regression analysis showed that non-disclosure of HIV status, previous tuberculosis treatment, ambulatory and bedridden functional status, and advanced WHO clinical stage were independent risk factors for early mortality. In our study, HIV status disclosure was found to be a predictor of prolonged survival in HIV patients on ART, in agreement with another study conducted in Addis Ababa, Ethiopia, where disclosure was found to have a protective effect for early mortality (aHR, 0.59; 95% CI, 0.36 to 0.96) [28]. Although future research may be necessary to establish the direct cause-and-effect association between non-disclosure status and early mortality in HIV patients, it is known that disclosure has more positive than negative social outcomes, such as improved social support, stronger family relationships, and reductions in anxiety and depression. Correspondingly, non-disclosure has detrimental health impacts associated with distress, loneliness, and medical non-adherence as a way to hide the presence of disease from others, and these factors may lead to a higher rate of mortality than in those who have disclosed their HIV status.

As in the present study, bedridden functional status (aHR, 5.91; 95% CI, 2.87 to 12.16) and advanced WHO stage (aHR, 7.36; 95% CI, 3.17 to 17.12) were reported to be independent predictors of mortality in the Somali region of Ethiopia [22], and in Addis Ababa among armed forces members [27]. The mortality incidence rate of 1.75 deaths per 100 person-years in the current study is in line with a study carried out in Debre-Markos, Ethiopia that reported 1.90 deaths per 100 person-years [19]; however, this rate was slightly lower than the mortality incidence rate of 2.03 deaths per 100 person-years reported in Southern Ethiopia [9] and East Ethiopia [32].

The current study found a much lower mortality incidence rate than early studies carried out in Ethiopia that reported mortality rates of 15.4 and 16.7 per 100 person-years in ART taking patients [33,34]. This indicates that ART is significantly reducing deaths among AIDS patients in Ethiopia and proves that the ART program is functioning well.

In conclusion, the probability of survival and the retention rate of patients on ART in this study was 64.0% and 79.0%, respectively, at 72 months of follow up, which is moderate and comparable with most results found in sub-Saharan countries. The independent predictors of mortality were non-disclosure of HIV status, previous tuberculosis treatment, ambulatory and bedridden functional status, and advanced WHO clinical stage. Hence, patients should be encouraged to disclose their status to their families or others, and those with an ambulatory or bedridden functional status and/or advanced WHO clinical stage disease should be monitored closely by their clinicians. Although hospital ART services are functioning well, further studies should investigate the causes of early mortality.

## Figures and Tables

**Table 1.** Socio-demographic characteristics of HIV patients receiving ART at Jinka Zonal Hospital, Jinka, South Omo, Ethiopia in 2016 (n=350)

Covariates	Category	Censored	Dead	Total (n)
Sex	Male	129 (90.2)	14 (9.8)	143
	Female	186 (89.9)	21 (10.1)	207
Marital status	Never	21 (80.8)	5 (19.2)	26
	Married	185 (89.8)	21 (10.2)	206
	Separated	42 (91.3)	4 (8.7)	46
	Widowed	20 (100.0)	0 (0.0)	20
	Divorced	47 (90.4)	5 (9.6)	52
Educational level	Illiterate	179 (87.7)	25 (12.3)	204
	Able to read and write	9 (100.0)	0 (0.0)	9
	Primary	98 (90.7)	10 (9.3)	108
	Secondary	23 (100.0)	0 (0.0)	23
	Tertiary	6 (100.0)	0 (0.0)	6
Occupation	Housewife	52 (91.2)	5 (8.8)	57
	Daily laborer	102 (89.5)	12 (10.5)	114
	Farmer	68 (87.2)	10 (12.8)	78
	Government employee	19 (86.4)	3 (13.6)	22
	Other	68 (93.2)	5 (6.8)	73
Religion	Orthodox	221 (98.5)	26 (10.5)	247
	Muslim	11 (84.6)	2 (15.4)	13
	Protestant	60 (93.8)	4 (6.2)	64
	Other	23 (88.5)	3 (11.5)	26
Residence type	Urban	223 (88.5)	24 (9.7)	247
	Rural	92 (89.3)	11 (10.7)	103
Age (yr)	18-30	180 (88.7)	23 (11.3)	203
	31-40	92 (92.9)	7 (7.1)	99
	41-50	31 (88.6)	4 (11.4)	35
	51-70	12 (92.3)	1 (7.7)	13

Values are presented as number (%). HIV, human immunodeficiency virus; ART, antiretroviral therapy.

**Table 2.** Baseline clinical and laboratory information of HIV patients receiving ART at Jinka Zonal Hospital, Jinka, South Omo, Ethiopia in 2016 (n=350)

Covariates	Category	Censored	Dead	Total (n)
Baseline weight (kg)	<60	237 (90.1)	26 (9.9)	263
	≥60	78 (89.7)	9 (10.3)	87
Baseline CD4 count (cells/μL)	10-200	105 (82.7)	22 (17.3)	127
	201-349	136 (92.5)	11 (7.5)	147
	350-499	57 (96.6)	2 (3.4)	59
	500-720	17 (100.0)	0 (0.0)	17
Functional status	Working	296 (93.1)	22 (6.9)	318
	Ambulatory	18 (69.2)	8 (30.8)	26
	Bedridden	1 (16.7)	5 (83.3)	6
WHO staging	I	54 (98.2)	1 (1.8)	55
	II	69 (92.0)	6 (8.0)	75
	III	171 (93.4)	12 (6.6)	183
	IV	21 (56.8)	16 (43.2)	37
ART eligibility criteria	WHO stage	47 (87.0)	7 (13.0)	54
	CD4 count	135 (91.8)	12 (8.2)	147
	Both	119 (88.1)	16 (11.9)	135
	Pregnancy	14 (100)	0 (0.0)	14
Substance use	Yes	119 (83.2)	24 (16.8)	143
	No	160 (96.4)	6 (96.4)	166
Opportunistic infection	No	36 (97.3)	1 (2.7)	37
	Yes	279 (89.1)	34 (10.9)	313

Values are presented as number (%). WHO, World Health Organization; ART, antiretroviral therapy; HIV, human immunodeficiency virus.

**Table 3.** Baseline characteristics and probability of survival during 6-year of follow-up (Kaplan-Meier method) of HIV patients receiving ART, Jinka Hospital, South Omo, Ethiopia in 2016 (n=350)

Characteristics	Mean survival probability over 6 yr and CI	p-value^1^
Past TB treatment		<0.001
Yes	50.86 (39.20, 62.57)	
No	65.29 (63.19, 67.39)	
Substance addiction		<0.001
Yes	59.71 (55.61,63.82)	
No	68.34 (66.25, 70.43)	
Functional status		< 0.001
Working	66.27 (64.39, 68.17)	
Ambulatory	46.46 (35.36, 57.55)	
Bedridden	13.25 (5.60, 20.90)	
WHO staging		0.03
I and II	67.12 (64.34, 69.89)	
III and IV	62.30 (59.03, 65.30)	
Baseline CD4 count (cells/μL)		0.03
10-350	52.83 (50.22, 65.43)	
351-1,200	68.84 (65.91, 71.76)	
Disclosure status		0.003
Yes	64.96 (62.83, 67.10)	
No	52.41 (40.03, 64.78)	
Opportunistic infections		0.13
Yes	66.19 (62.69, 69.70)	
No	63.45 (61.07, 65.84)	

HIV, human immunodeficiency virus; ART, antiretroviral therapy; CI, confidence interval; TB, tuberculosis; WHO, World Health Organization.

1 Log-rank test.

**Table 4.** Results of the bivariate and multivariate Cox regression analysis of HIV patients receiving antiretroviral treatment, Jinka Hospital, South Omo, Ethiopia in 2016 (n=350)

Characteristics	Bivariate HR (95% CI)	p-value	Multivariate aHR (95% CI)	p-value
Disclosure status				
Yes	1.00 (reference)		1.00 (reference)	
No	3.49 (1.48, 8.22)	0.004	5.82 (1.91, 17.72)	0.02
Past TB treatment				
Yes	4.20 (1.96, 8.99)	<0.001	1.82 (1.41,3.51)	0.04
No	1.00 (reference)		1.00 (reference)	
Functional status				
Working	1.00 (reference)		1.00 (reference)	
Ambulatory	5.98 (2.64, 13.54)	< 0.001	2.97 (1.20, 8.86)	0.05
Bedridden	20.64 (7.65, 55.68)	< 0.001	4.67 (1.30, 17.27)	0.02
WHO staging				
I and II	1.00 (reference)		1.00 (reference)	
III and IV	2.41 (1.50, 5.52)	0.04	3.25 (1.98, 10.7)	0.05
Baseline CD4 count (cells/μL)				
0-350	4.31 (1.34-17.97)	0.05	3.38 (0.71, 16.02)	0.33
351-1,200	1.00 (reference)		1.00 (reference)	
Substance use				
Yes	4.57 (1.87, 11.21)	0.001	3.72 (1.39, 9.97)	0.009
No	1.00 (reference)		1.00 (reference)	
Initial regimens				
1a=D4T-3TC-NVP	3.47 (1.24, 9.79)	0.02	1.66 (0.45, 6.14)	0.45
1b=D4T-3TC-EFV	2.95 (0.64, 13.71)	0.16	0.54 (0.07, 4.42)	0.57
1c=AZT-3TC-NVP	0.52 (0.07, 3.98)	0.53	0.39 (0.05, 3.28)	0.38
1d=AZT-3TC-EFV	0.00 (0.00, 0.00)	0.98	0.00 (0.00, 0.00)	0.98
1e=TDF-3TC-EFV	0.51 (0.24, 1.10)	0.08	0.20 (0.07, 0.53)	0.001
1f=TDF+3TC+NVP	1.00 (reference)		1.00 (reference)	

HR, hazard ratio; CI, confidence interval; aHR, adjusted hazard ratio; TB, tuberculosis; WHO, World Health Organization; AZT, zidovudine; 3TC, lamivudine; D4T, stavudine; NVP, nevirapine; EFV, efavirenz.

**Table 5.** Factors affecting ART retention rate and adverse outcomes of patients receiving ART at Jinka Zonal Hospital, Jinka, South Omo, Ethiopia in 2016 (n=350)

Covariate	Category	Adverse outcome	Retention	Crude OR (95% CI)	p-value	aOR (95% CI)	p-value
Marital status	Never	10 (38.5)	16 (61.5)	0.78 (0.29, 2.00)	0.61	0.78 (0.30, 0.50)	0.65
	Married	38 (18.4)	168 (81.6)	2.15 (1.10, 423)	0.03	2.07 (1.00, 4.40)	0.06
	Separated	8 (17.4)	38 (82.6)	2.31 (0.89, 6.01)	0.09	1.81 (0.70, 5.00)	0.25
	Widowed	10 (50.0)	10 (50.0)	1.00 (0.99, 1.34)	0.98	1.00 (0.90, 1.30)	0.30
	Divorced	17 (32.7)	35 (67.3)	1.00 (reference)		1.00 (reference)	
Occupation	Housewife	7 (12.3)	50 (87.7)	3.72 (1.47, 9.40)	0.005	3.34 (2.08, 18.48)	0.02
	Daily laborer	17 (14.9)	97 (85.1)	2.97 (0.80, 3.28)	0.003	2.60 (1.22, 5.52)	0.01
	Farmer	19 (24.4)	59 (75.6)	1.62 (0.80, 3.28)	0.18	1.50 (0.72, 3.37)	0.26
	Governmental employee	5 (22.7)	17 (77.3)	1.77 (0.59, 5.36)	0.31	1.80 (0.54, 6.00)	0.34
	Other	25 (34.2)	48 (65.8)	1.00 (reference)		1.00 (reference)	
WHO Staging	I	7 (12.7)	48 (83.3)	5.83 (2.10, 16.22)	0.001	6.20 (2.08, 18.48)	0.001
	II	13 (17.3)	62 (82.7)	4.05 (1.68, 9.78)	0.002	5.02 (1.94, 10.53)	0.001
	III	36 (19.7)	147 (80.3)	3.47 (1.65, 7.29)	0.001	4.63 (2.04, 10.53)	0.001
	IV	17 (45.9)	20 (54.1)	1.00 (reference)		1.00 (reference)	
							

Values are presented as number (%). ART, antiretroviral therapy; OR, odds ratio; aOR, adjusted odds ratio; CI, confidence interval; WHO, World Health Organization.
